# Supplementary material for: Structural Characterization, Cytotoxicity, and the Antifungal Mechanism of a Novel Peptide Extracted from Garlic (Allium sativa L.)
Source: Molecules. 2023 Mar 30;28(7):3098. doi: 10.3390/molecules28073098 (PMC10095746; doi:10.3390/molecules28073098)
Supplement: Supplementary file 1 [file molecules-28-03098-s001.zip › molecules-2293745-supplementary.pdf]

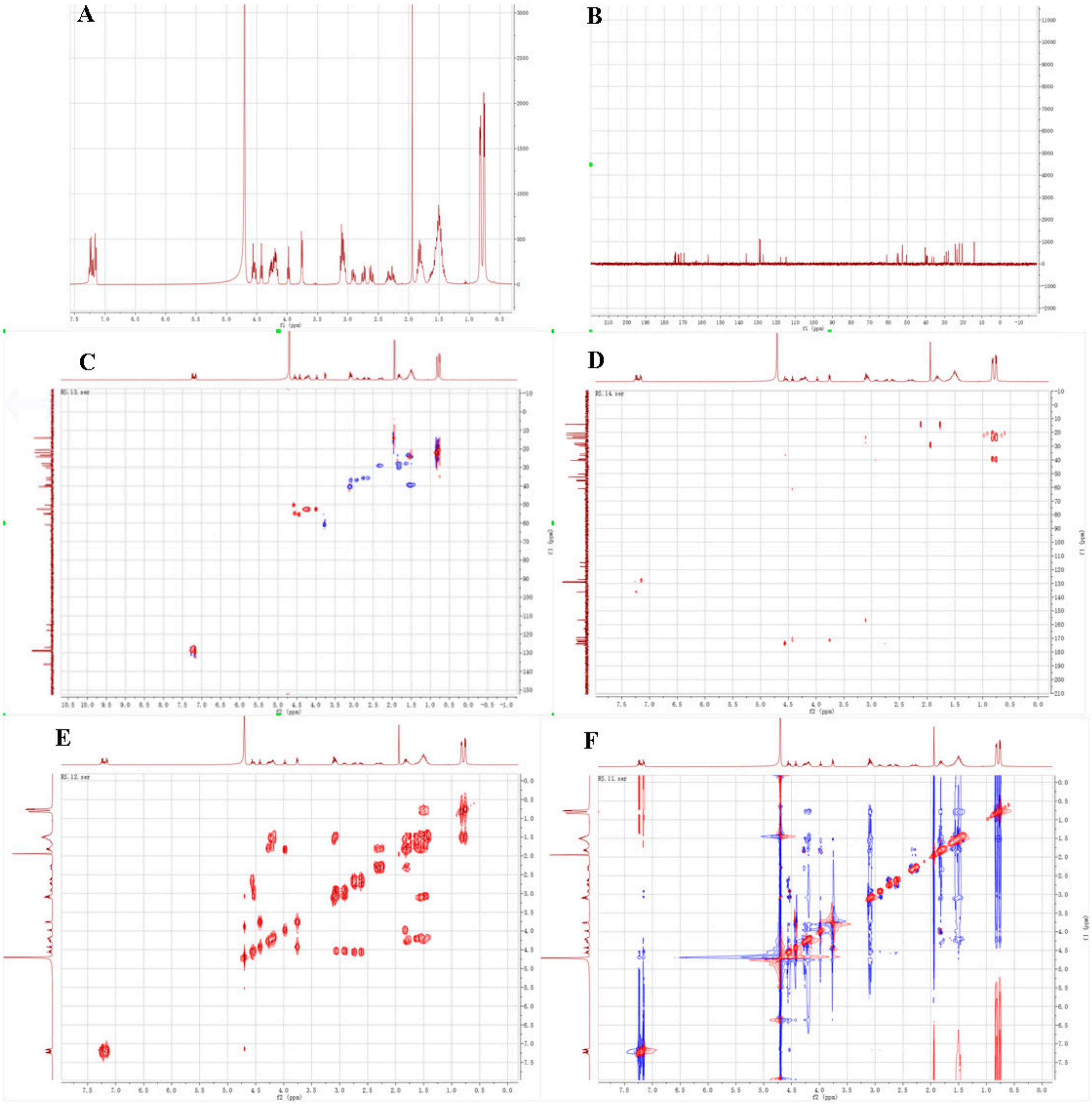


Figure S1 NMR spectra of RSLNLLMFR. A, $^{1}$H NMR spectrum. B, $^{13}$C NMR spectrum. C, HSQC NMR spectrum. D, HMBC NMR spectrum. E, COSY NMR spectrum. F, NOESY NMR spectrum
